# Characterization of an efficient N-oxygenase from *Saccharothrix* sp. and its application in the synthesis of azomycin

**DOI:** 10.1186/s13068-023-02446-5

**Published:** 2023-12-16

**Authors:** Chuanle Fan, Fang Zhou, Wei Huang, Yi Xue, Chao Xu, Rubing Zhang, Mo Xian, Xinjun Feng

**Affiliations:** 1 CAS Key Laboratory of Biobased Materials, Qingdao Institute of Bioenergy and Bioprocess Technology, Chinese Academy of Sciences, Qingdao, 266101 China; 2 Shandong Energy Institute, Qingdao, 266101 China; 3 Qingdao New Energy Shandong Laboratory, Qingdao, 266101 China

**Keywords:** Heme-oxygenase-like diiron oxygenase, Nitro compounds, Site-directed mutation, Whole-cell biocatalysis, Nitroimidazoles

## Abstract

**Background:**

The nitro group constitutes a significant functional moiety within numerous valuable substances, such as nitroimidazoles, a class of antimicrobial drugs exhibiting broad spectrum activity. Conventional chemical methods for synthesizing nitro compounds suffer from harsh conditions, multiple steps, and environmental issues. Biocatalysis has emerged as a promising alternative to overcome these drawbacks, with certain enzymes capable of catalyzing nitro group formation gradually being discovered in nature. Nevertheless, the practical application is hindered by the restricted diversity and low catalytic activity exhibited by the reported nitrifying enzymes.

**Results:**

A novel N-oxygenase SaRohS harboring higher catalytic capability of transformation 2-aminoimidazole to azomycin was characterized from *Saccharothrix* sp. Phylogenetic tree analysis revealed that SaRohS belongs to the heme-oxygenase-like diiron oxygenase (HDOs) family. SaRohS exhibited optimal activity at pH 5.5 and 25 ℃, respectively. The enzyme maintained relatively stable activity within the pH range of 4.5 to 6.5 and the temperature range of 20 ℃ to 35 ℃. Following sequence alignment and structural analysis, several promising amino acid residues were meticulously chosen for catalytic performance evaluation. Site-directed mutations showed that threonine 75 was essential for the catalytic activity. The dual mutant enzyme G95A/K115T exhibited the highest catalytic efficiency, which was approximately 5.8-fold higher than that of the wild-type and 22.3-fold higher than that of the reported N-oxygenase KaRohS from *Kitasatospora azatica*. The underlying catalytic mechanism was investigated through molecular docking and molecular dynamics. Finally, whole-cell biocatalysis was performed and 2-aminoimidazole could be effectively converted into azomycin with a reaction conversion rate of 42% within 14 h.

**Conclusions:**

An efficient N-oxygenase that catalyzes 2-aminoimidazole to azomycin was screened form *Saccharothrix* sp., its phylogenetics and enzymatic properties were analyzed. Through site-directed mutation, enhancements in catalytic competence were achieved, and the molecular basis underlying the enhanced enzymatic activity of the mutants was revealed via molecular docking and dynamic simulation. Furthermore, the application potential of this enzyme was assessed through whole cell biocatalysis, demonstrating it as a promising alternative method for azomycin production.

**Graphical Abstract:**

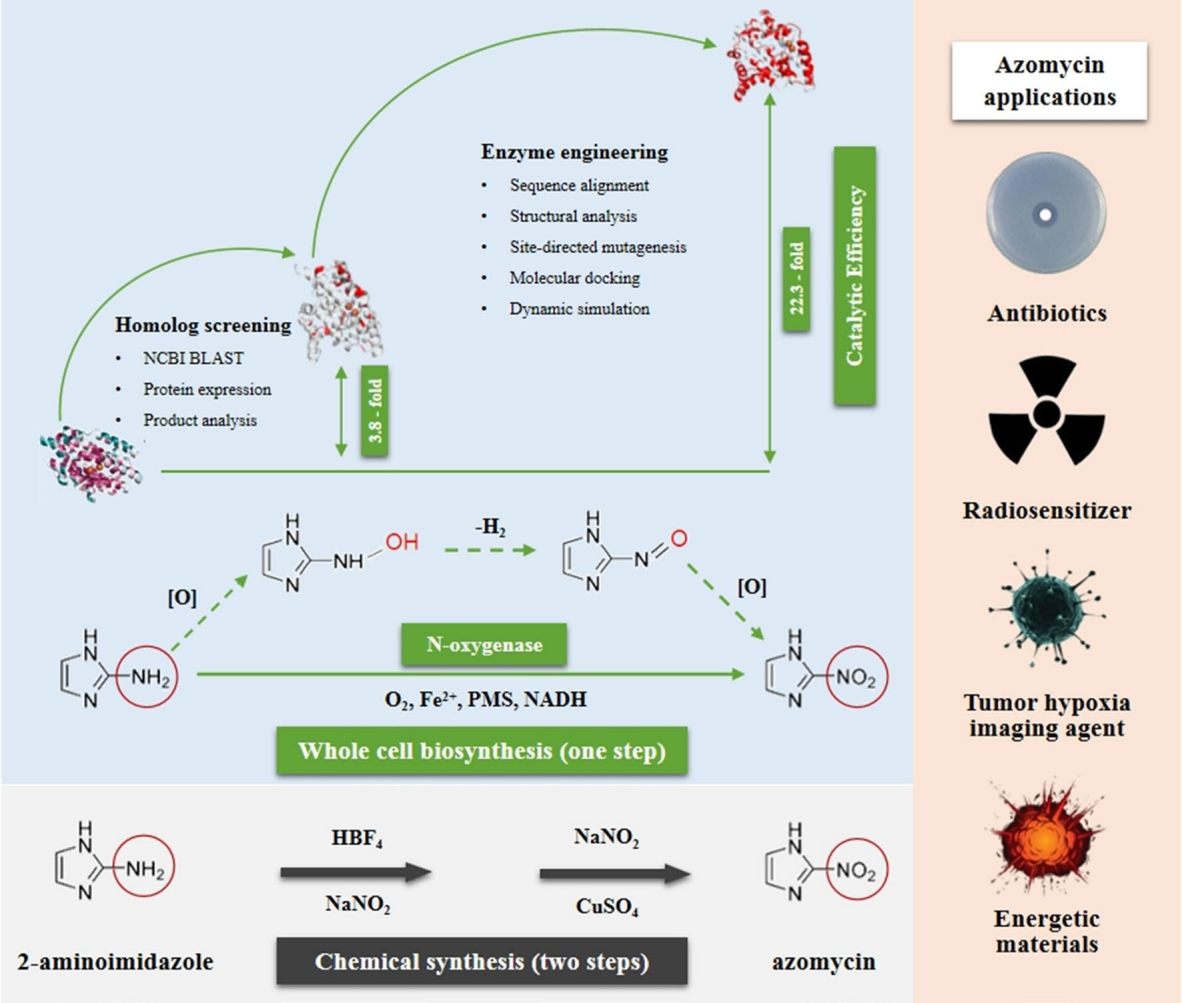

**Supplementary Information:**

The online version contains supplementary material available at 10.1186/s13068-023-02446-5.

## Background

The nitro group constitutes a significant functional moiety within numerous valuable substances such as pharmaceuticals, pigments and explosives [1]. Traditional chemical methods for synthesizing nitro compounds are dangerous and expensive with heavy environmental pollution [2]. Eco-friendly and less harmful methods are urgently needed. Biosynthetic nitration emerged as an attractive option due to its mild conditions, simple process and low environmental impact. To date, over 200 naturally nitro compounds were identified [3]. There are two main pathways that are conceivable for nitro compounds formation in nature: oxygenation of amines and direct nitration, and the N-oxidation of amines by N-oxygenases was considered as the most common strategy [4]. However, just few N-oxygenases, such as AurF that catalyzes the oxidation of the amino group of *p*-aminobenzoate to a nitro group in aureothin biosynthesis [5], PrnD that catalyzes the formation of nitropyrrolnitrin from aminopyrrolnitrin in pyrrolnitrin biosynthesis [6], and CmlI that catalyzes the NH_2_-CAM precursor to the aryl-nitro group of chloramphenicol [7], have been characterized. Despite its significant importance, there are only a limited number of characterized N-oxygenases. Hence, there is an urgent need to characterize more enzymes for nitro group formation.

Nitroimidazoles are an exceptional class of antimicrobial drugs known for their extraordinary broad spectrum activity against anaerobic bacteria, parasites, and mycobacteria [8]. 2-nitroimidazole, also named azomycin, was the first nitroimidazole antibiotics isolated from culture filtrates of *Streptomyces eurocidicus* [9]. Azomycin has been shown to have effective activity against *Trichomonas vaginalis* [10], utility as radiosensitizers in tumour treatment [11], and it is also a potential precursor for the synthesis of dinitro- or trinitroimidazole explosives [12]. Many attempts to synthesize azomycin had initially failed, and it was eventually achieved through diazotization of 2-aminoimidazole following with the Gattermann reaction, 10 years after its discovery [13]. This chemical synthesis method was carried out using large amounts of strong inorganic acid (HCl, HBF_4_) and organic solvent (ethyl acetate, ethanol) with a relatively low yield of about 30%.

Some early studies revealed that L-arginine can be converted into azomycin via the intermediate precursor of 2-aminoimidazole in *S. eurocidicus* [14, 15]. However, it was not until 2019 that the biosynthetic enzymes responsible for azomycin production were finally identified. Hedges and Ryan [16] identified the biosynthetic gene cluster and reconstituted the enzymatic steps from l-arginine to azomycin in vitro. In this pathway, l-arginine was converted to 2-aminoimidazole step-by-step by O_2_-, PLP-dependent arginine oxidase RohP, retro-aldolase RohR and cyclodehydratase RohQ. And RohS from *Kitasatospora azatica* (KaRohS) was identified as a N-oxygenase which catalyzed the oxidation of 2-aminoimidazole to azomycin when phenazine methosulfate (PMS), NADH, and FeSO_4_ were present. However, its catalytic rate was only 5% of that of AurF and PrnD.

In our study, a new and efficient N-oxygenase SaRohS that catalyzes the conversion of 2-aminoimidazole to azomycin was identified in *Saccharothrix* sp. To better understand its biological properties, a thorough analysis encompassing phylogenetics and enzymatic properties have been conducted. Furthermore, the catalytic efficiency was enhanced by rational design based on sequence comparison and structure simulation analysis, and the molecular basis for increased catalytic efficiency of the mutants was revealed through molecular docking. Finally, the catalytic capability for azomycin production was assessed by whole cell biocatalysis with the dual sites mutant enzyme G95A/K115T.

## Results and discussion

### Screening of efficient N-oxygenase

NCBI BLAST search was conducted with the sequence of the reported KaRohS (WP_051969388.1) [16] as template. Closely related homologs were identified and sequences with alignment > 60% (over the length of the protein) were considered (Additional file 9: Table S1). The comparison results showed that most of the sequences were originated from *Streptomyces* sp. and it is known that their corresponding proteins easily precipitate during in vitro reactions [16], so they were not considered for screening in this study. Except for the aforementioned, one sequence from each of the genera *Kitasatospora*, *Pseudonocardia*, *Actinokineospora*, and *Saccharothrix* (with approximately 59% similarity to RohS) were selected for catalytic performance assessment (Table 1). These four genes were separately ligated into plasmid pETDuet-28a and expressed in *E.coli* BL21 (DE3) cells. All the genes are well expressed in the bacteria and the corresponding proteins were purified for in vitro assays (Additional file 1: Fig. S1). The production of azomycin was confirmed by LC–MS analysis (Additional file 2: Fig. S2), and HPLC analysis allowed the determination and product concentration was determined by HPLC analysis to compare the catalytic ability. AtRohS and SaRohS showed a stronger capability of concerting 2-aminoimidazole to azomycin than the control trial, which enriched the sources of N-oxygenase. The highest concentration of azomycin was obtained when SaRohS was used, which was 3.6-fold higher than that of KaRohS.

**Table 1:** The selected homologous gene information and in vitro azomycin production

Name	Species	Indent (%)	Accession	Azomycin (μM)
KaRohS	*Kitasatospora azatica*	100	WP_051969388.1	4.2
KvRohS	*Kitasatospora viridis*	80.53	WP_145907253.1	trace amount
PbRohS	*Pseudonocardiales bacterium*	77.78	MBV8540531.1	0
AtRohS	*Actinokineospora terrae*	64.07	WP-092782860.1	12.2 ± 0.4
SaRohS	*Saccharothrix sp.*	59.07	NUT99080.1	15.2 ± 0.7

### Bioinformatics analysis of SaRohS

The N-oxygenase SaRohS is derived from *Saccharothrix* sp., with a gene of 846 base pairs that encodes 281 amino acids. This gene can be identified by its Genbank sequence number Nut99080.1. ProtParam analysis of SaRohS protein suggests that its theoretical isoelectric point (pI) value is 4.83 and the molecular weight is 31.2 KDa. Positively charged residues (Arg + Lys) and negatively charged residues (Asp + Glu) in the sequence were found to be 26 and 40, respectively. The computed instability index (II) is 36.7, indicating that the enzyme is stable under natural conditions (Protein with II values < 40 is considered stable). Moreover, the grand average of hydropathicity (GRAVY) value is –0.005, indicating that SaRohS is hydrophilic protein (Protein with negative GRAVY value is hydrophilic while it is considered hydrophobic with positive GRAVY value).

### Phylogenetic analysis

**Fig. 1:**
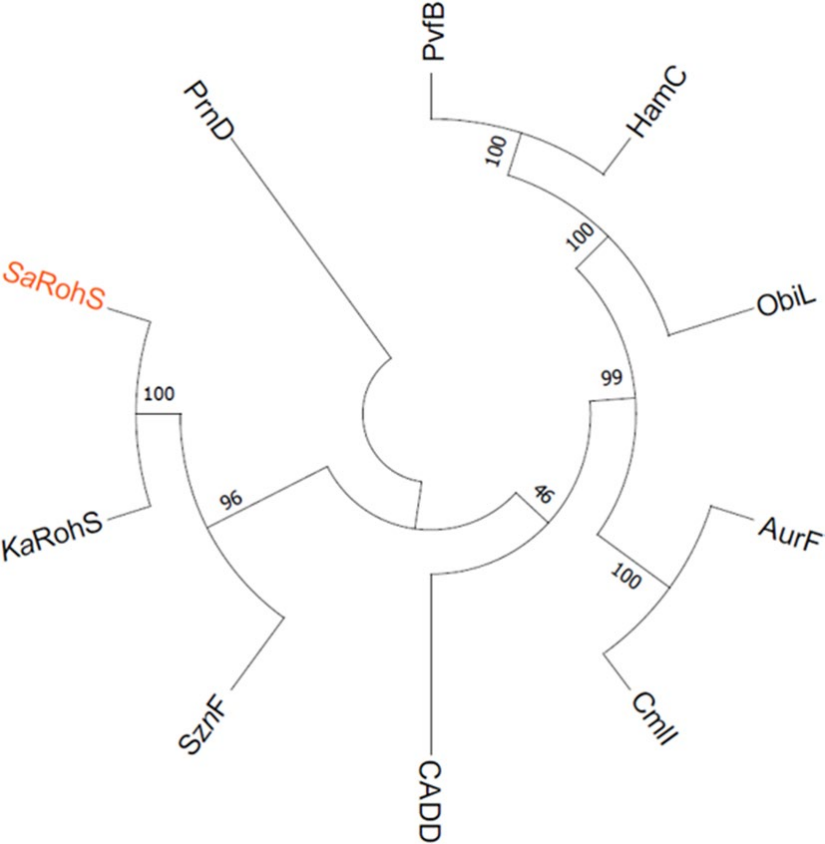
Phylogenetic tree of SaRohS. The amino acid sequences of the above enzymes were compared by Mega 11 software version 11.0.11. GenBank ID: SaRohS, NUT99080.1; KaRohS, WP_051969388.1; AurF, CAE02601.1; PrnD, AEL33291.1; SznF, QBA82202.1; PvfB, CAK13110.1; CADD, 1RCW_C; CmlI, CCA54211.1; ObiL, ARJ35757.1; HamC, ABK09932.1

Phylogenetic tree analysis (Fig. 1) was performed to determine the relationship between SaRohS and the reported N-oxygenase. The results showed that SaRohS is distinct from the FDO (ferritin-like diiron oxidase and oxygenase) superfamily such as AurF [17], CmlI [18], ObiL [19], PvfB [20] that belong to the Pfam 11583. Similar to KaRohS, SaRohS is closely related to the heme-oxygenase-like diiron oxygenase (HDO) SznF [21], which falls under Pfam 14518. Although bioinformatic analysis suggests that ~ 9600 HDOs exist in nature, just four of them have undergone biochemical characterization: N-oxygenase SznF and KaRohS, desaturase/lyases UndA [22] and BesC [23]. The large majority of unexplored HDOs hold enormous potential for new reactivities. Even though both SznF and KaRohS are classified as N-oxygenase, they actually exhibit a distinct oxidation functions. SznF was the first reported N-oxygenase to generate the N-hydroxylation of two distinct nitrogen atoms within a single molecule, which catalyzes the formation of N^δ^-hydroxy-N^ω^’-methyl-N^ω^-nitroso-L-citrulline, a crucial precursor of streptozotocin, from N^ω^-methyl-N^ω^-L-arginine. KaRohS, considering its ability to catalyze the oxidation of 2-aminoimidazole into its nitro derivative, seems closer to the FDO superfamily enzymes of AurF, CmlI, and PrnD.

### Effect of pH, temperature and organic solvent on enzyme activity

**Fig. 2:**
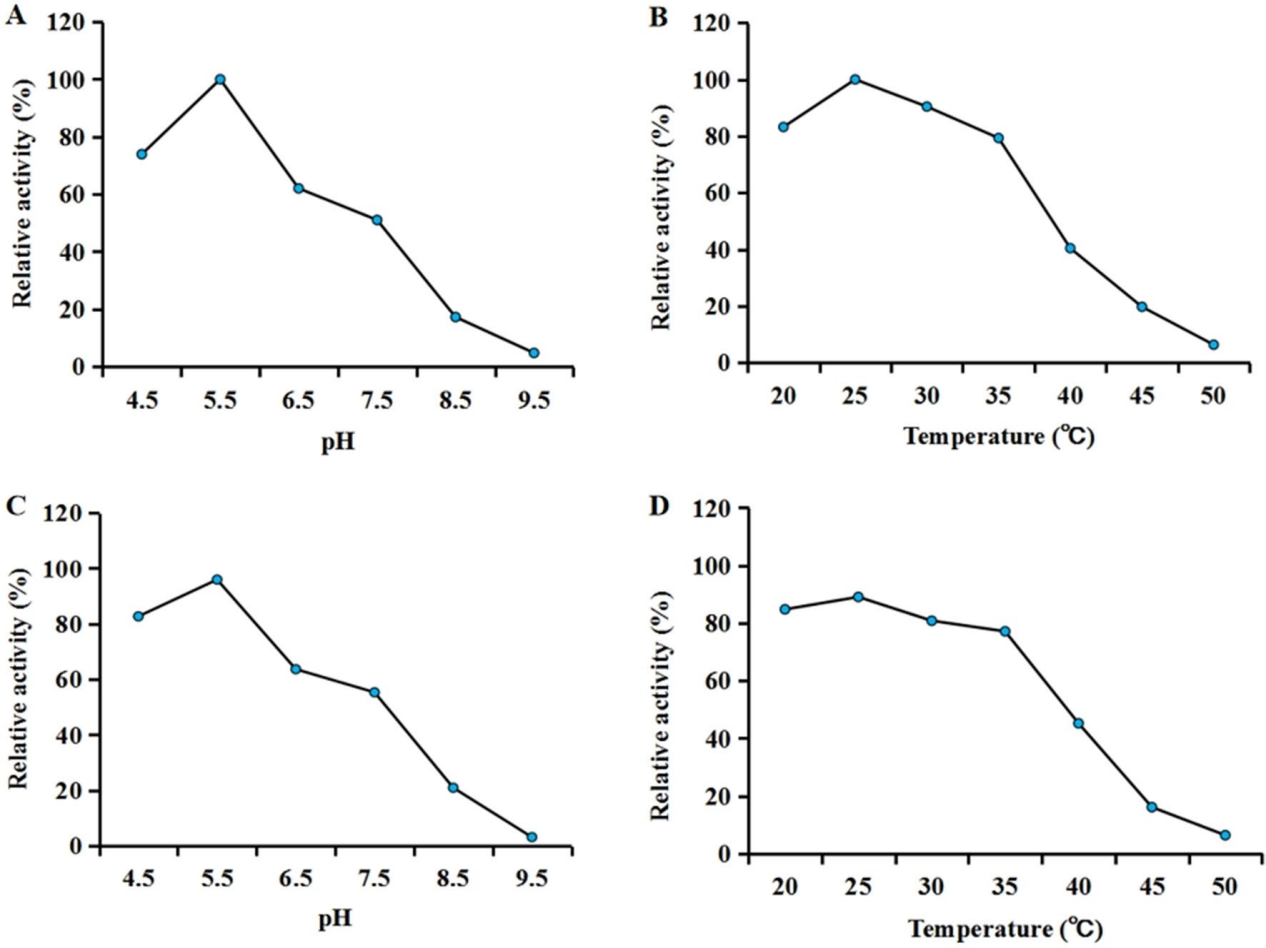
Enzymatic properties of SaRohS. **A** Determination of the optimum pH of SaRohS. **B** Determination of the optimum temperature of SaRohS. **C** Stability of SaRohS protein under different pH conditions. **D** Stability of SaRohS protein at different temperatures. The stability of the enzyme was determined after incubation for 1 h under different conditions

To explore the optimum pH and temperature of SaRohS, the enzyme’s activity was tested under varying pH levels and temperatures. As shown in Fig. 2A, the relative activity of SaRohS reached its peak at pH 5.5 close to the isoelectric point and decreased significantly when the pH exceeds this value. The enzyme activity was only 51%, 17%, 4.7% of the maximum activity at pH7.5, 8.5, and 9.5, respectively. In addition to the adaptability of SaRohS, the increased activity at lower pH may be attributed to more favorable protonation/redox state when the PMS/NADH reduction system is used [24]. The effect of temperature on the enzyme activity is shown in Fig. 2B. Over the temperature range of 20 ℃ to 35 ℃, the relative activity can consistently remain above 80%. The optimum temperature of SaRohS was found to be 25 ℃ which was also employed for KaRohS [16]. When the temperature rise to 40 ℃, the enzyme activity plummeted to only 40% of its maximum level. Further, at a temperature of 50 ℃, the enzyme was nearly completely inactivated, with only 6% of its activity remaining.

The stability was evaluated after incubation for 1 h under various conditions. SaRohS exhibited good stability between pH 4.5 and 6.5, and the activity decreased significantly when the pH exceeded 6.5 (Fig. 2C). At a pH of 9.5, the residual enzyme activity was merely 3.1%. The thermostability of purified SaRohS was also tested over the range of temperatures from 20 °C to 50 °C (Fig. 2D). The enzyme activity remained stable below 40 °C and gradually decreased as the temperature rose to 40 °C. Only 6.4% of the residual activity was detected after an incubation of 1 h at 50 °C.

The influence of various organic solvents on the stability of SaRohS was also investigated. Unfortunately, the protein showed a low organic solvents tolerance (Additional file 9: Table S2). Even 20% v/v of acetonitrile, dichloromethane, chloroform, and *n*-butanol completely deactivated the enzyme. Moreover, it was noted that 20% v/v methanol, ethanol, DMSO, and ethyl acetate led to approximately 51%, 34%, 52%, and 72% reduction in activity, respectively, followed by complete enzyme inactivation in the presence of 50% v/v methanol, ethanol, and DMSO.

### Prediction of mutation sites and activity assay

**Fig. 3:**
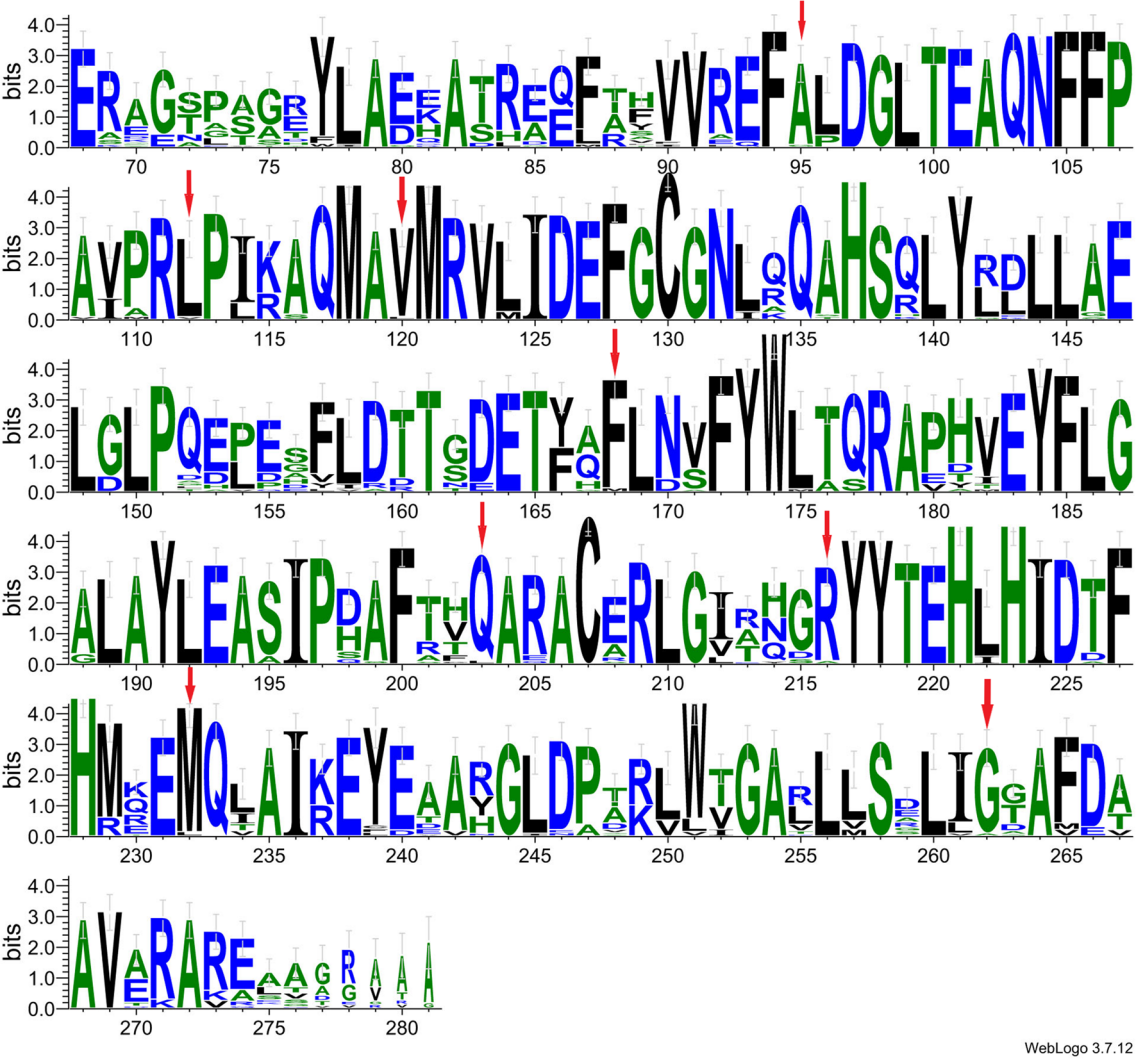
The sequence logo of N-oxygenase sequences with residue numbers corresponding to SaRohS. 30 hypothetical proteins have been collected. SequenceLogo was generated using WebLogo 3.7.12. The selected sites for mutation are marked with red arrows

Nowadays, a variety of methods, including sequence-based, structure-based, and the more new artificial intelligence-based approaches, have been employed to enhance and design the enzyme performance. To predict the key amino acid residues, an initial multiple amino acid sequence alignment was performed between SaRohS and 30 hypothetical proteins, which shared more than 60% identity. Multiple sequence alignment was analyzed using the ClustalW method. The Sequence logo has been prepared using WebLogo with an equiprobable reference amino acid composition, comparison of amino acid sites from 68 to 281 (Fig. 3). Eight amino acid residues were found conserved in all proteins except SaRohS: G95A, V112L, L120V, M168F, L205Q, A216R, L232M, and A262G, which may play an important role in the enzyme activity. To obtain more potential sites, three-dimensional structure analysis of SaRohS was also performed. Due to the lack of knowledge about the protein's structure, homology model was built using RoseTTAFold. Meanwhile, the definitely conserved sites, relatively conserved sites, and variable sites were analyzed by the Consurf server (Additional file 3: Fig. S3). Based on the predicted structure information, five amino acid sites of T75, K115, S198, L212, and D266 among the variation sites were meticulously chosen for further evaluation (Table 2). For example, the substrate channel of the protein might undergo enlargement upon mutation of L212, located at the bottom of the cavity formed by the α-helical bundle, to either V212 or I212, and consequently could affect the protein’s catalytic activity.

**Table 2:** Analysis of the proposed sites for site-directed mutagenesis based on structural simulation

Proposed sites	Mutation information	Annotation
T75	A75	T75 was located adjacent to the α-helical bundle. When the hydrophilic amino acid threonine was replaced with the hydrophobic amino acid alanine, the steric hindrance could be reduced and the substrate channel could enlarged, ultimately affect the catalytic activity of the protein
K115	R115, T115	The side-chain of K115 is oriented outward. After the lysine was mutated to either arginine or threonine, the orientation could change and subsequently affect the steric hindrance
S198	D198, Y198	After S198 was mutated to D198 or Y198, additional interaction may be created with other amino residue, such as A69, and consequently affect the protein’s structure
L212	V212, I212	L212 was located at the bottom of the cavity formed by the α-helical bundle. The mutation of L212 to either V212 or I212 may result in enlargement of the substrate channel of the protein, consequently affect its catalytic activity
D266	K266, A266	D266 is hydrogen bonded to S74. After D266 was mutated to K266 or A266, the hydrogen bond between them disappeared. This loss of the hydrogen bond may increase the flexibility and enlarge the substrate channel

**Fig. 4:**
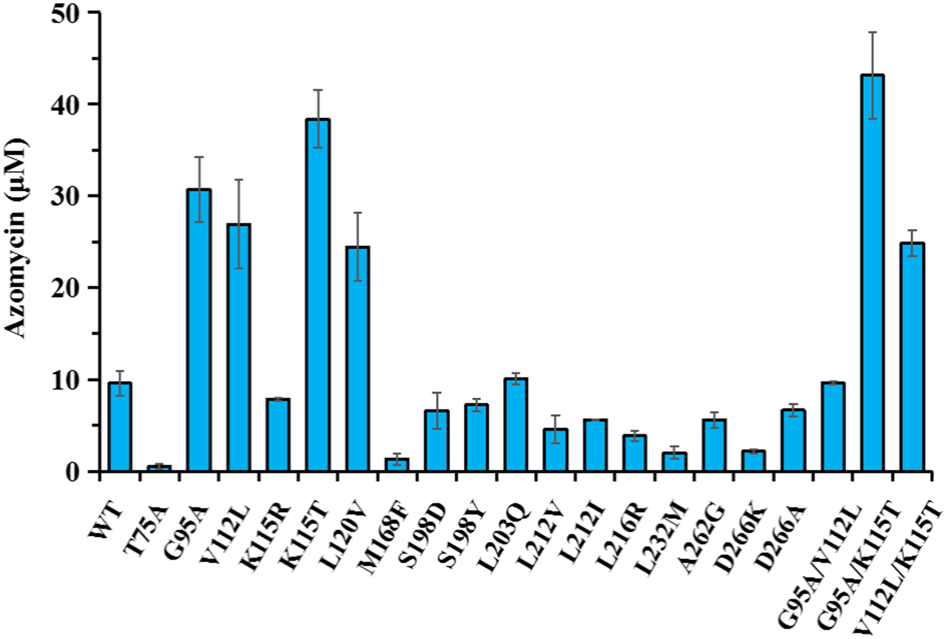
Effect of SaRohS mutagenesis on azomycin production in vitro. Data were obtained after each reaction was induced for 20 min. All the experiments were carried out in triplicate

A total of 17 single mutants were constructed and expressed in vivo, with the aim of comparing their relative activities to that of the wild-type SaRohS in in vitro assays. As shown in Fig. 4, the product concentration was significantly increased in the presence of mutant enzymes G95A, V112L, K115T or L120V. Remarkably, K115T mutant achieved the highest catalytic capacity, which was fourfold of that of the original enzyme. In contrast, mutations at T75, M168, L232, or D266 sites resulted in a noticeable reduction in production. More strikingly, the introduction of the T75A mutation caused the product to decrease to less than 5% of its original level, indicating the vital role of threonine 75 in the catalytic activity. To further improve the enzymatic capacity, the effect of the dual mutants G95A/V112L, G95A/K115T, V112L/K115T on azomycin production were also evaluated. The mutant G95A/K115T exhibited the best azomycin synthesis efficiency, which was 4.5-fold of that of the wild type.

Kinetic parameters (*k*_*cat*_ and *K*_*m*_) were obtained by fitting the data to the Michaelis–Menten equation, and the catalytic efficiency was determined as *k*_*cat*_/*K*_*m*_. As shown in Table 3, the dual mutant enzyme G95A/K115T exhibited a significantly higher catalytic efficiency for 2-aminoimidazole conversion (*k*_*cat*_/*K*_*m*_ = 13.4 × 10^–3^ µM^−1^ min^−1^), which was approximately 22.3-fold greater than that of the reported KaRohS (*k*_*cat*_/*K*_*m*_ = 0.6 × 10^–3^ µM^−1^ min^−1^) and 5.8-fold higher than that of the wild-type SaRohS (*k*_*cat*_/*K*_*m*_ = 2.3 × 10^–3^ µM^−1^ min^−1^). Additionally, the G95A/K115T mutant demonstrated an increased affinity of approximately 4.7-fold and 2.2-fold over that of KaRohS and SaRohS, respectively. Moreover, the catalytic rate of the G95A/K115T mutant was also increased (*k*_*cat*_ ~ 1.11 min^−1^ compared to 0.23, 0.42 min^−1^ for the other two enzyme). Meanwhile, the mutant T75A displayed a very low catalytic efficiency in line with the azomycin production.

**Table 3:** Kinetic parameters of SaRohS and important mutants compared with KaRohS

	KaRohS	SaRohS	T75A	G95A/K115T
*k*_*cat*_ (min^−1^)	0.23 ± 0.005	0.42 ± 0.007	0.051 ± 0.003	1.11 ± 0.005
*K*_*m*_ (µM)	390.55 ± 0.02	182.69 ± 0.01	924.17 ± 0.10	82.68 ± 0.001
*k*_*cat*_/*K*_*m*_ (µM^−1^ min^−1^)	0.6 × 10^–3^	2.3 × 10^–3^	0.06 × 10^–3^	13.4 × 10^–3^

### Analysis of the mechanism of SaRohS and its mutants with increased activity

**Fig. 5:**
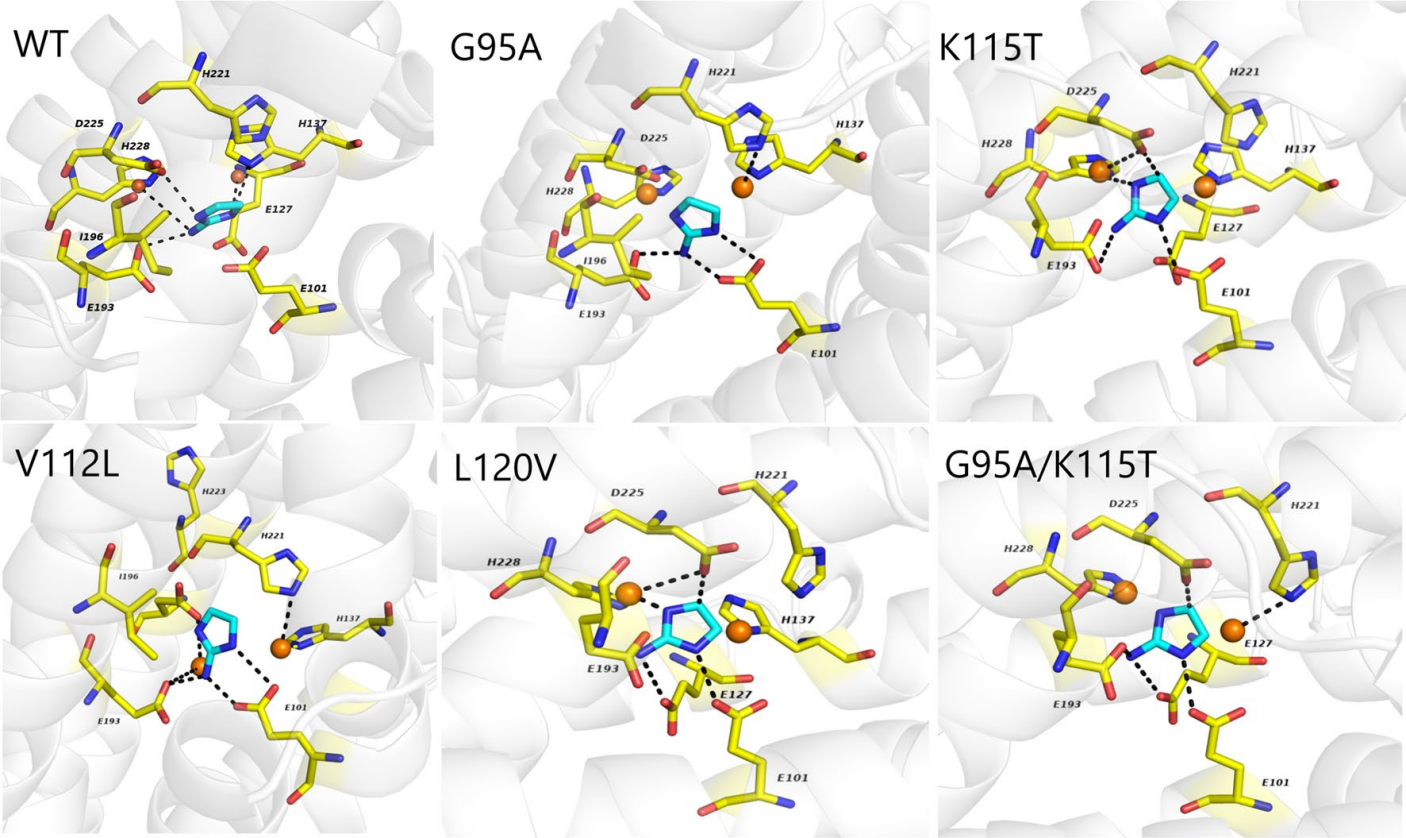
The enzyme–substrate molecular docking results of wild SaRohS and each mutants using GOLD software. The yellow lines represent the residues in SaRohS, 2-aminoimidazole are brilliant blue and the black dashed line represents a hydrogen bond

To elucidate the molecular basis of the enhanced activity of SaRohS and its mutants, the 3D structure of the enzyme was predicted and docked with the substrate 2-aminoimidazole. The enzyme–substrate molecular interaction network is shown in Fig. 5 and Additional file 4: Fig. S4. It was observed that the variants exhibited more interactions with the substrate compared to the wild type. For instance, an additional van der Waals force was found between G95A/K115T and 2-aminoimidazole. Meanwhile, the binding free energy calculated using MM/PBSA was also found to be higher for the variants compared to the wild type (Additional file 9: Table S3). Specifically, the G95A/K115T mutant displayed a binding free energy of − 190.58 kcal/mol, whereas the wild type had a value of  − 115.06 kcal/mol. These observed phenomenons indicated an increased affinity, which was consist with the *K*_*m*_ analysis.

The conformation changes were also analyzed using the molecular dynamic simulation method. Remarkably, all the variants exhibited lower RMSD values compared to the wild type which means that they have good protein stability (Additional file 5: Fig. S5, Additional file 6: Fig. S6, Additional file 7: Fig. S7). And the flexibility of the catalytic active center increased after the specific amino acid sites mutated. Specifically, the rigidity of the 100–140 amino acid residues region increased after G95 mutated to A95 combination with K115 mutated to T115, thereby promoting a more stable structure. On the contrary, the dual mutant of G95A/K115T increased the flexibility of the three amino acids V109, M229 and K230 located in the catalytic active center, good flexibility can facilitate the substrate catalysis and may be the reason for the improved catalytic efficiency. Because research on RohS is recent and limited, little is known about its structure, including the active sites. Although the results obtained through structural simulation and molecular docking provide some useful information for revealing the catalytic mechanism, the real crystal structure is necessary to an in-depth study in the future.

### Whole-cell synthesis of azomycin

**Fig. 6:**
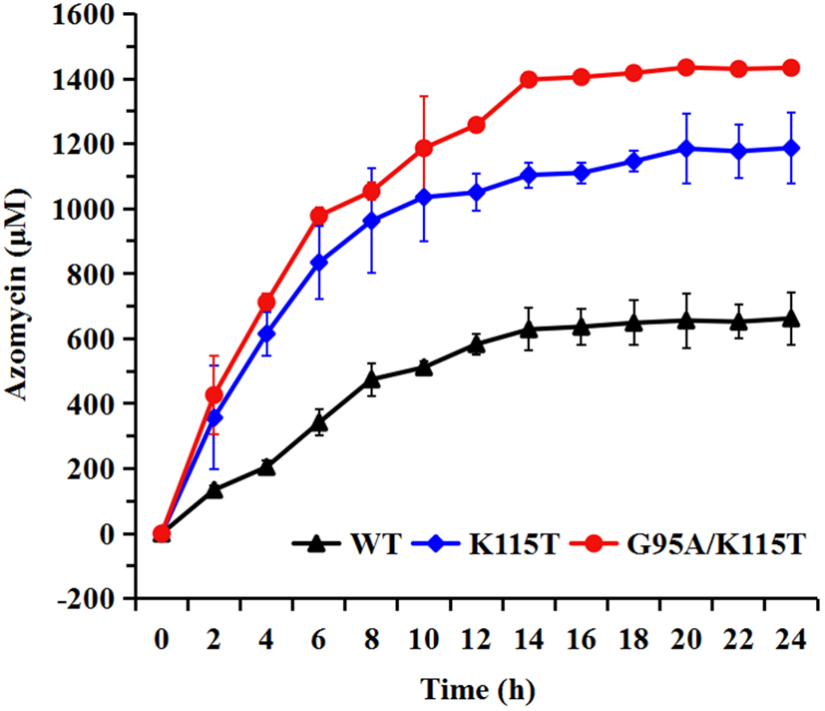
The time profiles of azomycin accumulation of whole-cell synthesis performed in a 200-mL bioreactor. Wild type with black line, K115T mutant with blue line, and G95A/K115T mutant with red line

To further evaluate the application prospects of the screened and modified N-oxygenase, whole-cell synthesis experiments were carried out in a 200 mL bioreactor. As the whole-cell catalysis results showed (Fig. 6, Additional file 8: Fig. S8), SaRohS and its variants possess high catalytic efficiency for 2-aminoimidazole. For instance, when the dual mutant G95A/K115T was employed, the conversion of substrate into intermediates with the final product and the reaction conversion rate of azomycin was significantly higher compared to the reported N-oxygenase. Specifically, within a span of 14 h, the conversion reached an impressive 83%, while the reaction conversion rate reached 42%. This is the most efficient process described to date and offers a promising alternative method for azomycin production.

The emerging HDO family appears to have a unifying trait, which is an unstable oxidized cofactor [25]. This distinct characteristic could also promote product release, thus preventing further oxidation at the central domain and allowing the transfer to the cupin domain to complete the catalytic process leading to a final nitroso product. Although the characteristic mechanism of RohS has yet to be fully understood, it can be speculated that the arylamine to arylnitro transformation proceeded step by step with some intermediates rather than all in one fell swoop. This assumption can be used to explain the phenomenon of the low azomycin production synchronous with high 2-aminoimidazole conversion rate, a certain intermediates may exist and cannot translate effectively to the next step which limit the final yield. What's more, like some well-studied genuine N-oxygenases AurF [17], CmlI [18], PrnD [26], the intermediates may be a hydroxylamine and a nitroso product during the whole catalytic process of RohS which is not analyzed in this study. The complete catalytic mechanism is urgent to reveal, which will be beneficial to enzyme modification and process optimization for future practical applications.

## Conclusions

This study successfully screened and characterized a novel N-oxygenase from *Saccharothrix* sp., which possesses a higher catalytic competency in transformation 2-aminoimidazole to azomycin compared to the reported N-oxygenase KaRohS. Phylogenetic tree analysis revealed that SaRohS belongs to the HDOs family. SaRohS exhibited optimal activity at pH 5.5 and 25 ℃, respectively. The enzyme maintained relatively stable activity within the pH range of 4.5 to 6.5 and temperature range of 20 ℃ to 35 ℃. Through sequence alignment and structure analysis, several promising amino acid residues were meticulously chosen for catalytic performance evaluation. Site-directed mutations demonstrated that five amino acid residues greatly influence the catalytic performance. Specifically, the mutant T75A almost completely lost catalytic activity for 2-aminoimidazole, indicating that this site was essential for the catalytic activity. G95A, V112L, K115T and L120V demonstrated their importance to the higher catalytic activity. The dual mutant enzyme G95A/K115T showed the highest catalytic efficiency, which was approximately 5.8-, 22.3-fold higher than that of the wild-type SaRohS and the reported KaRohS, respectively. The underlying catalytic mechanism was analyzed via molecular docking and molecular dynamics, and some important information was displayed. Finally, whole-cell biocatalysis was performed and proved a promising alternative method for azomycin production.

## Materials and methods

### Strains, plasmids and primers

All the strains, plasmids and primers used in this study are listed in Additional file 9: Table S4. The *E. coli* DH5α strain (Invitrogen) was used to construct and amplify vectors. The *E.coli* BL21 (DE3) was used for recombinant proteins expression and azomycin production. The homologous genes *rohS*_Kv_, *rohS*_Pb_, *rohS*_At_, and *rohS*_Sa_ were codon optimized and chemically synthesized by Beijing Liuhe BGI, and cloned into the plasmid pETDuet-28a^+^ between EcoRI and BamHI sites to construct recombinant plasmids. Mutations were introduced into the *rohS*_Sa_ gene using a one-step PCR method. All the recombinant plasmids were verified by colony PCR and nucleotide sequencing.

### Prokaryotic expression and protein purification

To express recombinant proteins, a single colony of *E. coli* BL21 (DE3) containing the expression plasmid was incubated overnight in LB medium with 50 μg/mL kanamycin at 37 °C and 200 rpm. The culture was then diluted 1:100 into fresh LB medium. Once the cell density reached approximately 0.6–0.8 OD_600_, protein expression was induced by adding 0.2 mM β-d-1-thiogalactopyranoside (IPTG), followed by further growth for 16 h at 16 °C. The cells were collected and washed with HEPES buffer (20 mM HEPES, 50 mM NaCl, and 10 mM imidazole, pH 7.5). After the washing process, the cells were resuspended in 1 mL buffer and introduced into the high pressure cell disrupter. Subsequently, the mixture was centrifuged at a speed of 12,000 rpm for 45 min. The supernatant containing the recombinant proteins was purified by Ni–NTA His-bind Resin column. Initially, the column was equilibrated with 1 × binding buffer (20 mM HEPES, 50 mM NaCl, and 10 mM imidazole). Following sample loading, the impure proteins were removed with wash buffer (20 mM HEPES, 50 mM NaCl, and 20 mM imidazole). The target protein was then eluted using elution buffer (20 mM HEPES, 50 mM NaCl, and 250 mM imidazole). Finally, the purified protein was confirmed using SDS-PAGE and quantified via the BSA standard curve method.

### In vitro biochemical analysis

The in vitro reaction was carried out in a 1 mL reaction system containing 2 mM 2-aminoimidazole, 10 μM RohS, 2 mM FeSO_4_·7H_2_O, 50 μM phenazine methosulfate (PMS), and 5 mM NADH, in 20 mM HEPES buffer. The reactions proceeded for 20 min at 25 ℃ and were quenched with addition of an equal volume of MeOH. The precipitate was removed by centrifugation, and 10 μL of the resulting supernatant was used to verify azomycin production by HPLC and LC–MS.

### Enzymatic properties

To detect the effect of temperature and pH on the enzyme activity, the in vitro reaction was carried out at different temperature (25 °C, 30 °C, 35 °C, 40 °C, 45 °C, 50 °C) and different pH (4.5, 5.5, 6.5, 7.5, 8.5, 9.5) for 20 min. The activity of RohS at the optimum temperature and pH was used as control, the relative activity under different conditions was calculated with the control as 100%. For determination of Michaelis–Menten kinetic parameters, the reaction was carried out with the concentration of 2-aminoimidzole varied from 1 to 5 mM and initiated with addition of 10 μM RohS into the reaction system. The reaction proceeded for 20 min at pH 5.5, 25 ℃ and was quenched with addition of MeOH.

### Enzyme stability

The purified SaRohS was incubated under different temperatures (20 °C, 25 °C, 30 °C, 35 °C, 40 °C, 45 °C, and 50 °C) and pH values (4.5, 5.5, 6.5, 7.5, 8.5, 9.5) for 1 h, respectively. Then, 10 μM enzyme, 2 mM 2-aminoimidazole, 2 mM FeSO_4_·7H_2_O, 50 μM PMS, and 5 mM NADH were added successively, and the reaction time was 20 min at 25 °C with pH 5.5. The activity of SaRohS at the optimum temperature and pH was used as a blank control, and the determination method was the same as above. The residual activity was calculated with the blank control as 100%.

The stability of SaRohS in organic solvents was assessed by measuring the remaining activity after pre-incubating the buffer containing 25 and 50% (v/v) of various solvents at 25 °C with an agitation speed of 200 rpm for 20 min. The final enzyme concentrations were adjusted to 10 μM before the pre-incubation. Both water-miscible and water-immiscible organic solvents, including methanol, ethanol, acetonitrile, dimethylsulfoxide (DMSO), dichloromethane, chloroform, *n*-butanol, and ethyl acetate, were tested on the enzyme. Control mixtures without any organic solvent were included. The residual activity was calculated as a percentage of the relative activity compared to the control mixture, with the control set at 100%.

### Analysis of physicochemical properties

The physicochemical properties of protein SaRohS were analyzed using the Expasy ProtParam server (https://web.expasy.org/protparam/). EXPASY ProtParam gives a detailed result about the molecular weight, theoretical isoelectric point (PI), the total number of positively and negatively charged residues, the instability index, and grand average of hydropathicity (GRAVY) [27].

### Phylogenetic analysis

The genomic sequences of KaRohS, AurF, PrnD, SznF, PvfB, CADD, CmlI, ObiL, and HamC were downloaded from GenBank. Phylogenetic analysis was conducted by Mega 11 software version 11.0.11. The phylogenetic tree was constructed by the neighbor-joining method. The percentage of replicate trees in which the associated taxa clustered together in the bootstrap test (2000 iterations) was calculated. The similarity of amino acid sequences was compared by the NCBI website.

### Site-directed mutagenesis

Using pETDuet-28a^+^-*rohS*_Sa_ as a template, the primers containing the mutation sites were designed and PCR amplification was conducted using the Fast Mutagenesis V2 Kit (Vazyme, China, Nanjing). PCR products were digested with *Dpn*I to eliminate the template plasmid and then transferred to *E. coli* DH5α for amplification, sequencing, and the successfully mutated plasmid was transferred to the expression strain *E. coli* BL21 (DE3) to obtain the mutants.

### Homology modeling and molecular docking

The cupin domain-containing diiron protein (Protein Data Bank [PDB]: 6vzy) was used as a template to calculate the Fe coordinates of SaRohS through the protein website SWISS-MODEL (https://swissmodel.expasy.org/). The 3D structural model of SaRohS was created using the Protein structure prediction software RoseTTAFold. The binding of 2-aminoimidazole in SaRohS was simulated using the molecular docking program GOLD. Gold_kinase_VS template was applied and the maximum number of generic algorithm runs was set to 100. GoldScore was chosen as a fitness function and the standard default settings were employed in all calculations.

### Whole-cell synthesis reactions

The induced cells were resuspended with 50 mL HEPES buffer to obtain a density of 20 OD_600_, and the reaction system was consisted of 4 mM 2-aminoimidazole, 2 mM FeSO_4_·7H_2_O, 50 μM PMS, and 5 mM NADH. The reaction was carried out at 25 °C, pH 5.5 in a 200 mL bioreactor, and the agitation speed was kept at a constant rate of 200 rpm during 24 h. The samples were withdrawn every 2 h to determine residual 2-aminoimidazole and azomycin production. The reaction conversion rate of azomycin was calculated as the ratio of the actual production to the theoretical production.

### Analytic methods

The cell density at OD_600_ was measured via an ultraviolet spectrophotometer (Varian Cary 50 UV–Vis, US) to monitor cell growth. The samples were obtained by centrifugation of the reaction mixture at 10,000×*g* for 10 min, then filtered through a 0.22 μm Tuffryn membrane. The filtered samples were analyzed by HPLC using an Agilent 1260 HPLC apparatus equipped with a Luna C18, 5 μm, 4.6 mm ID × 250 mm column (Phenomenex). Elution was performed at a flow rate of 0.5 mL·min^−1^ using a mobile-phase consisting of a linear gradient of water and acetonitrile ((v/v):90:10, 0 to 5 min, 90:10 to 0:100, 5 to 6 min; 0:100, 6 to 10 min, 90:10, 10 to 11 min; 90:10 11 to 15 min), with both solvents containing 0.05% (v/v) trifluoroacetic acid. For 2-aminoimidazole detection, the elution was performed at a flow rate of 0.5 mL·min^−1^ using a mobile-phase consisting of a linear gradient of water and methanol ((v/v): 95:5 to 85:15, 0 to 5 min; 85:15 to 75:25, 5 to 15 min; 0:100, 20 to 25 min; 0:100 to 95: 5, 25 to 35 min; 95:5 35 to 40 min), with both solvents containing 5 mM ammonium acetate. The LC–MS analysis was conducted using a Ulimate 3000 UHPLC system coupled to a Compact Q-TOF LC/MS system, performed in positive ion mode with a mass scan range of 30 to 800 and an ESI ion source.

### Supplementary Information

**Additional file 1: Figure S1.** Verification of the proteins’ expression and purification by SDS-PAGE.

**Additional file 2: Figure S2.** Verification of the azomycin product by different enzymes using LC–MS. A, the specific ion flow of the standard and production with different enzymes; B, the mass spectrum of the azomycin standard; C, the mass spectrum of the azomycin production with KaRohS; D, the mass spectrum of the azomycin production with SaRohS; E, the mass spectrum of the azomycin production with AtRohS; F, the mass spectrum of the azomycin production with KvRohS.

**Additional file 3: Figure S3.** The predicted 3D structure of SaRohS (A). The definitely conserved sequence was colored with red, the relatively conserved sequence was colored with green, and the variation sequence was colored with white. The selected sites for site-directed mutation of T75, K115, D198, L212, D266 were displayed as B, C, D, E, and F, respectively.

**Additional file 4: Figure S4.** The enzyme–substrate molecular interaction network of wild SaRohS and each mutants. Conventional hydrogen bond with bright green, Van del Waals with medium green, Carbon hydrogen bond with light green, Metal acceptor with grey, Pi-alkyl with pink, Pi-sigma with purple, unfavorable acceptor–acceptor with red, Pi-anion with brown.

**Additional file 5: Figure S5.** Dynamic simulation of G95A mutant. A, Root-mean-square deviation (RMSD) value; B, Root-mean-square fluctuation (RMSF) value; C, Radius of gyration (Rg) value; D, the amino acid residues with increased flexibility after mutation; E, the conformation change of the substrate in the catalytic center, the blue stick is the conformation before mutation and the white stick is the conformation after mutation.

**Additional file 6: Figure S6.** Dynamic simulation of K115T mutant. A, RMSD value; B, RMSF value; C, Rg value; D, the amino acid residues with increased flexibility after mutation; E, the conformation change of the substrate in the catalytic center, the blue stick is the conformation before mutation and the white stick is the conformation after mutation.

**Additional file 7: Figure S7.** Dynamic simulation of G95A/K115T mutant. A, RMSD value; B, RMSF value; C, Rg value; D, the amino acid residues with increased flexibility after mutation; E, the conformation change of the substrate in the catalytic center, the blue stick is the conformation before mutation and the white stick is the conformation after mutation.

**Additional file 8: Figure S8.** The profile of the residual 2-aminoimidazole during the whole-cell synthesis with G95A/K115T mutant.

**Additional file 9: Table S1.** Information of proteins given > 60% sequence identity with KaRohS. **Table S2.** Organic solvent tolerance of SaRohS. **Table S3.** The binding free energy calculated using MM/PBSA. **Table S4** Strains, plasmids and primers used in this study.

## Data Availability

We provide all the necessary data for the publication of this article. All additional data are present in the article and the additional material documents.

## Abbreviations

*E. coli*: *Escherichia coli*
*S. eurocidius*: *Streptomyces eurocidius*
PI: Isoelectric point
HDO: Heme-oxygenase-like diiron oxygenase
FDO: Ferritin-like diiron oxidase and oxygenase
GRAVY: Grand average of hydropathicity
RMSD: Root-mean-square deviation
LC–MS: Liquid chromatography–mass spectrometer
HPLC: High performance liquid chromatography
OD_600_: Optical density at wavelength 600 nm
IPTG: Isopropyl-β-d-thiogalactoside
PMS: Phenazine methosulfate
