# Supplementary figures and images for: Characterization of an efficient N-oxygenase from Saccharothrix sp. and its application in the synthesis of azomycin
Source: Biotechnol Biofuels Bioprod. 2023 Dec 16;16:194. doi: 10.1186/s13068-023-02446-5 (PMC10724926; doi:10.1186/s13068-023-02446-5)

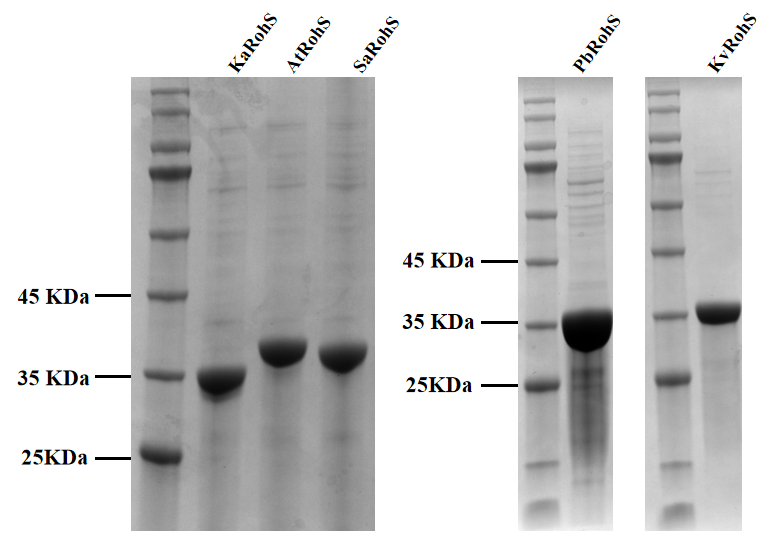

Supplement: Supplementary file 1 — Additional file 1: Figure S1. Verification of the proteins’ expression and purification by SDS-PAGE. [file 13068_2023_2446_MOESM1_ESM.png]

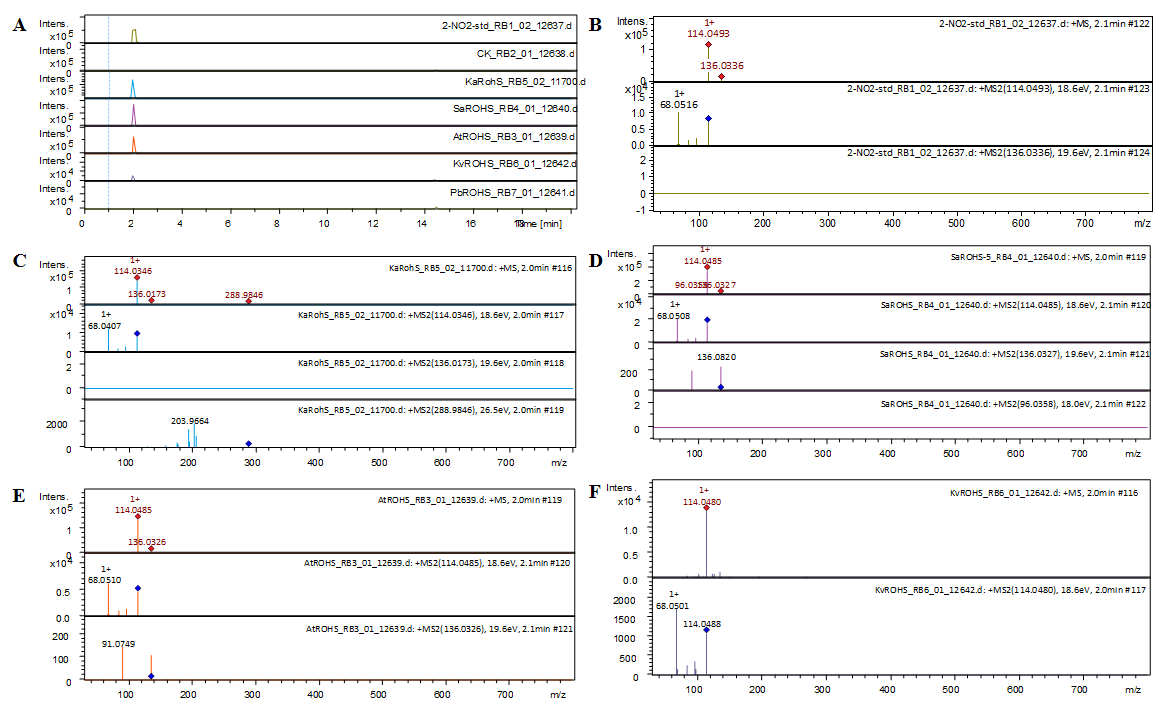

Supplement: Supplementary file 2 — Additional file 2: Figure S2. Verification of the azomycin product by different enzymes using LC–MS. A, the specific ion flow of the standard and production with different enzymes; B, the mass spectrum of the azomycin standard; C, the mass spectrum of the azomycin production with KaRohS; D, the mass spectrum of the azomycin production with SaRohS; E, the mass spectrum of the azomycin production with AtRohS; F, the mass spectrum of the azomycin production with KvRohS. [file 13068_2023_2446_MOESM2_ESM.png]

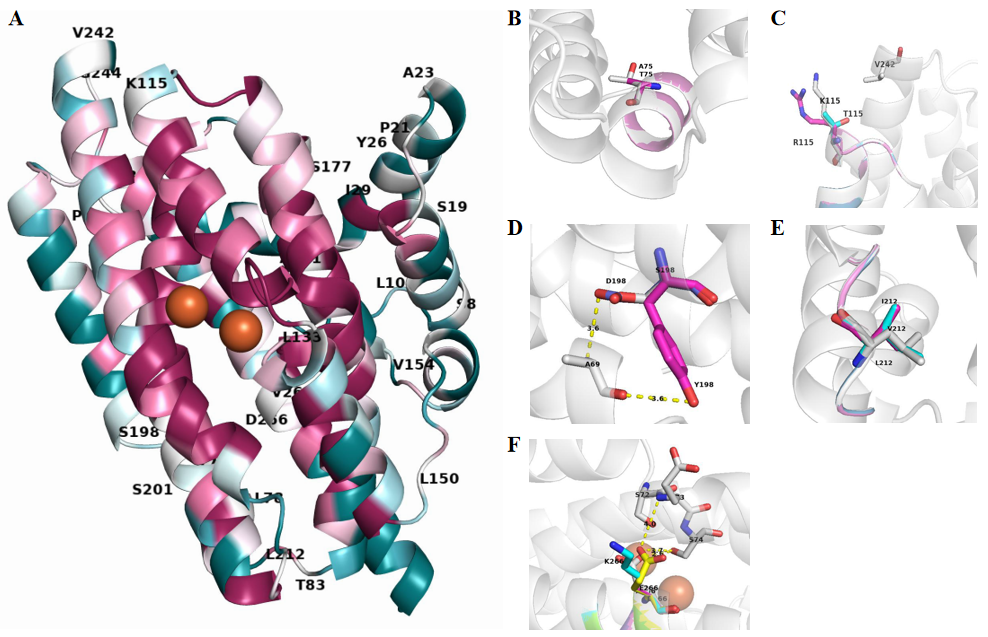

Supplement: Supplementary file 3 — Additional file 3: Figure S3. The predicted 3D structure of SaRohS (A). The definitely conserved sequence was colored with red, the relatively conserved sequence was colored with green, and the variation sequence was colored with white. The selected sites for site-directed mutation of T75, K115, D198, L212, D266 were displayed as B, C, D, E, and F, respectively. [file 13068_2023_2446_MOESM3_ESM.png]

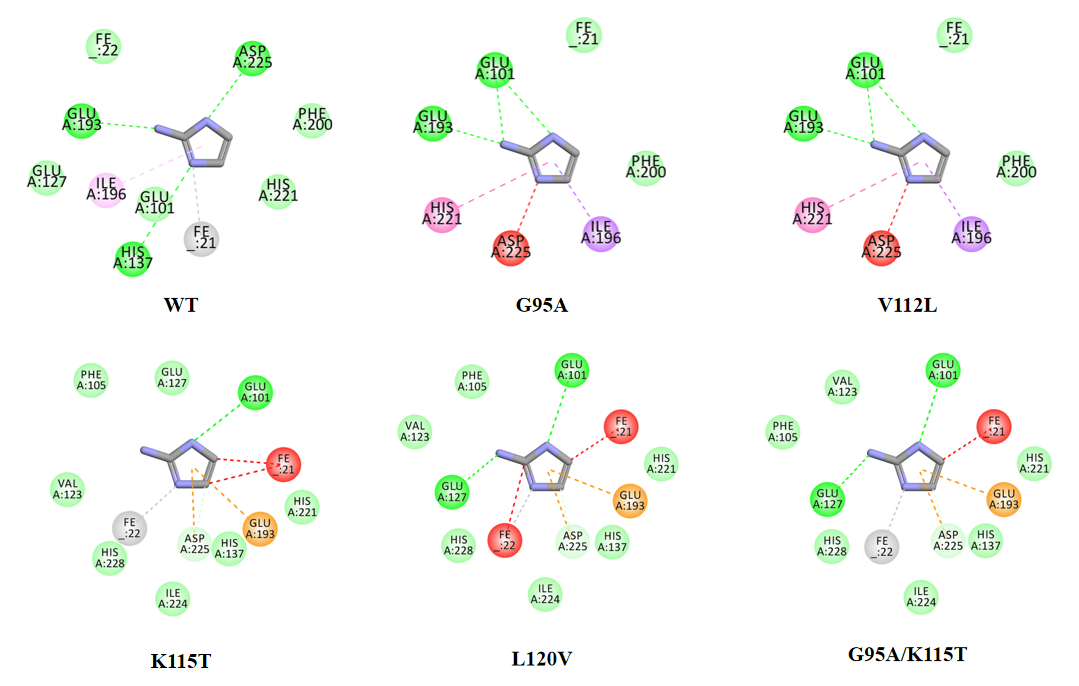

Supplement: Supplementary file 4 — Additional file 4: Figure S4. The enzyme–substrate molecular interaction network of wild SaRohS and each mutants. Conventional hydrogen bond with bright green, Van del Waals with medium green, Carbon hydrogen bond with light green, Metal acceptor with grey, Pi-alkyl with pink, Pi-sigma with purple, unfavorable acceptor–acceptor with red, Pi-anion with brown. [file 13068_2023_2446_MOESM4_ESM.png]

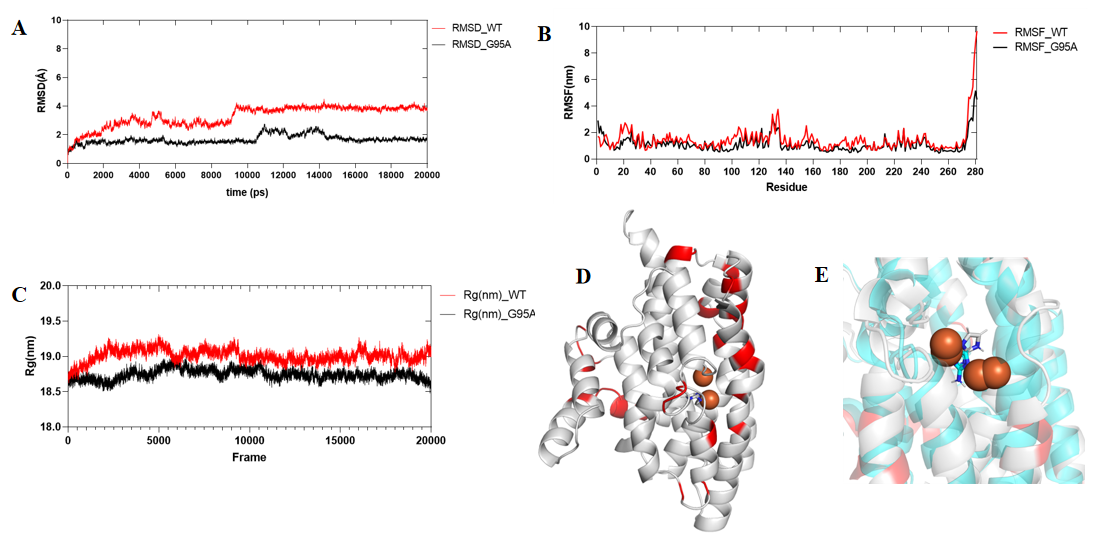

Supplement: Supplementary file 5 — Additional file 5: Figure S5. Dynamic simulation of G95A mutant. A, Root-mean-square deviation (RMSD) value; B, Root-mean-square fluctuation (RMSF) value; C, Radius of gyration (Rg) value; D, the amino acid residues with increased flexibility after mutation; E, the conformation change of the substrate in the catalytic center, the blue stick is the conformation before mutation and the white stick is the conformation after mutation. [file 13068_2023_2446_MOESM5_ESM.png]

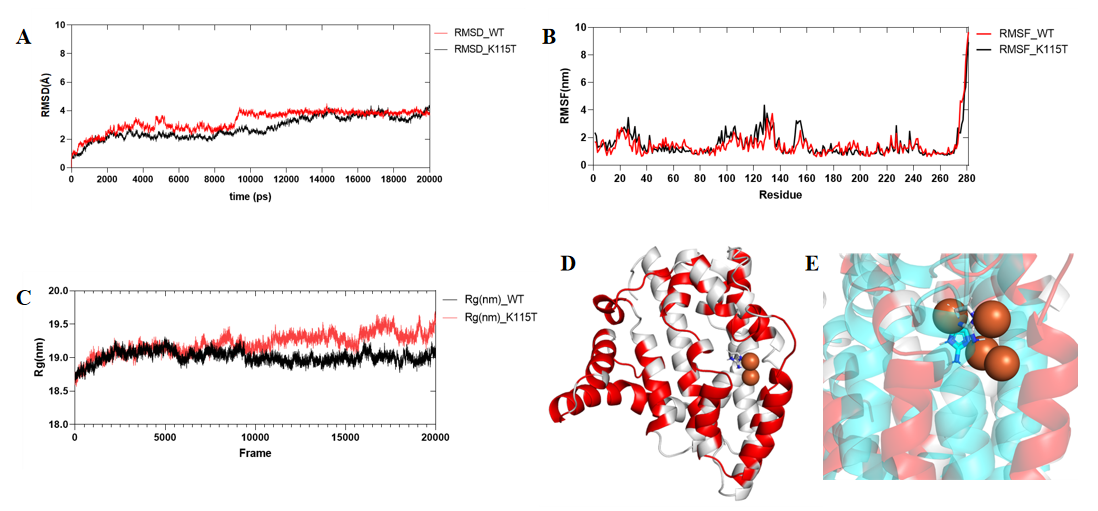

Supplement: Supplementary file 6 — Additional file 6: Figure S6. Dynamic simulation of K115T mutant. A, RMSD value; B, RMSF value; C, Rg value; D, the amino acid residues with increased flexibility after mutation; E, the conformation change of the substrate in the catalytic center, the blue stick is the conformation before mutation and the white stick is the conformation after mutation. [file 13068_2023_2446_MOESM6_ESM.png]

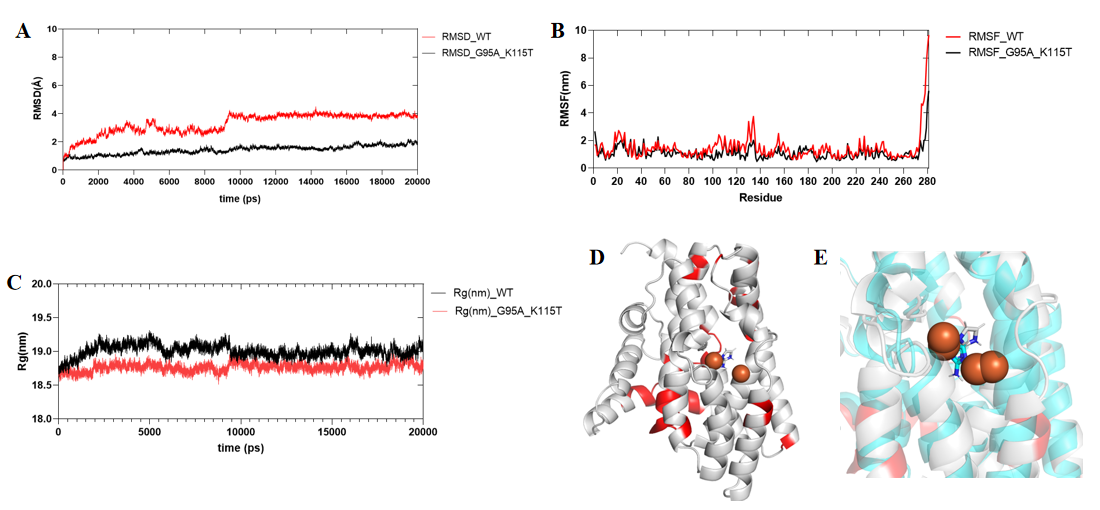

Supplement: Supplementary file 7 — Additional file 7: Figure S7. Dynamic simulation of G95A/K115T mutant. A, RMSD value; B, RMSF value; C, Rg value; D, the amino acid residues with increased flexibility after mutation; E, the conformation change of the substrate in the catalytic center, the blue stick is the conformation before mutation and the white stick is the conformation after mutation. [file 13068_2023_2446_MOESM7_ESM.png]

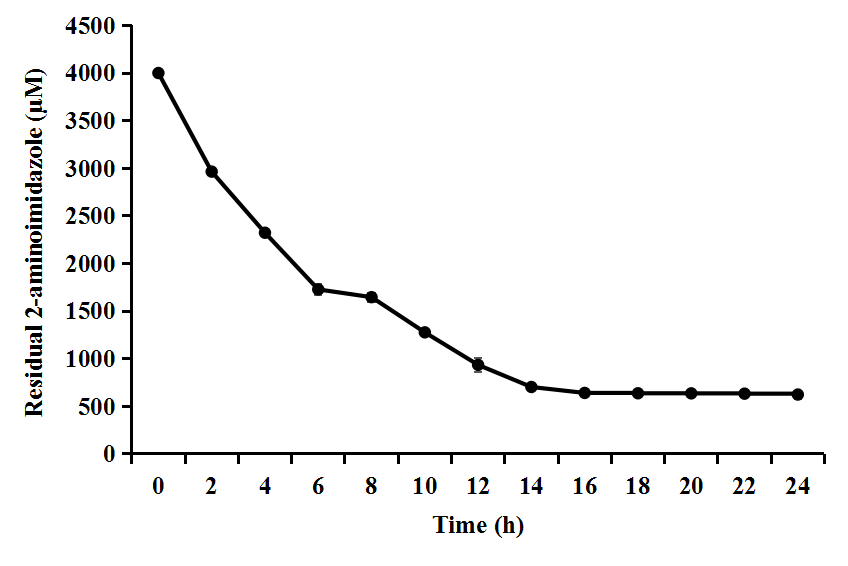

Supplement: Supplementary file 8 — Additional file 8: Figure S8. The profile of the residual 2-aminoimidazole during the whole-cell synthesis with G95A/K115T mutant. [file 13068_2023_2446_MOESM8_ESM.png]
