# Supplementary material for: Characterization of an efficient N-oxygenase from Saccharothrix sp. and its application in the synthesis of azomycin
Source: Biotechnol Biofuels Bioprod. 2023 Dec 16;16:194. doi: 10.1186/s13068-023-02446-5 (PMC10724926; doi:10.1186/s13068-023-02446-5)
Supplement: Supplementary file 9 — Additional file 9: Table S1. Information of proteins given > 60% sequence identity with KaRohS. Table S2. Organic solvent tolerance of SaRohS. Table S3. The binding free energy calculated using MM/PBSA. Table S4 Strains, plasmids and primers used in this study. [file 13068_2023_2446_MOESM9_ESM.docx]

**Figure captions**

**Figure S1:** Verification of the proteins’ expression and purification by SDS-PAGE.

**Figure S2:** Verification of the azomycin product by different enzymes using LC-MS. A, the specific ion flow of the standard and production with different enzymes; B, the mass spetrum of the azomycin standard; C, the mass spetrum of the azomycin production with KaRohS; D, the mass spetrum of the azomycin production with SaRohS; E, the mass spetrum of the azomycin production with AtRohS; F, the mass spetrum of the azomycin production with KvRohS.

**Figure S3:** The predicted 3D structure of SaRohS (A). The definitely conserved sequence was colored with red, the relatively conserved sequence was colored with green, and the variation sequence was colored with white. The selected sites for site-directed mutation of T75, K115, D198, L212, D266 were displayed as B, C, D, E, and F, respectively.

**Figure S4:** The enzyme-substrate molecular interaction network of wild SaRohS and each mutants. Conventional hydrogen bond with bright green, Van del waals with medium green, Carbon hydrogen bond with light green, Metal acceptor with grey, Pi-alkyl with pink, Pi-sigma with purple, unfavorable acceptor-acceptor with red, Pi-anion with brown.

**Figure S5:** Dynamic simulation of G95A mutant. A, Root-mean-square deviation (RMSD) value; B, Root-mean-square fluctuation (RMSF) value; C, Radius of gyration (Rg) value; D, the amino acid residues with increased flexibility after mutation; E, the conformation change of the substrate in the catalytic center, the blue stick is the conformation before mutation and the white stick is the conformation after mutation.

**Figure S6:** Dynamic simulation of K115T mutant. A, RMSD value; B, RMSF value; C, Rg value; D, the amino acid residues with increased flexibility after mutation; E, the conformation change of the substrate in the catalytic center, the blue stick is the conformation before mutation and the white stick is the conformation after mutation.

**Figure S7:** Dynamic simulation of G95A/K115T mutant. A, RMSD value; B, RMSF value; C, Rg value; D, the amino acid residues with increased flexibility after mutation; E, the conformation change of the substrate in the catalytic center, the blue stick is the conformation before mutation and the white stick is the conformation after mutation.

**Figure S8:** The profile of the residual 2-aminoimidazole during the whole-cell

synthesis with G95A/K115T mutant.

**Table S1** Information of proteins given >60% sequence identity with KaRohS.

| Accession | Description | Query Cover | Per. ident | aa_length |
| --- | --- | --- | --- | --- |
| WP_051969388.1 | iron-containing redox enzyme family protein [*Kitasatospora azatica*] | 100% | 100 | 286 |
| WP_255925465.1 | iron-containing redox enzyme family protein [*Streptomyces rubrisoli*] | 94% | 80.81 | 277 |
| WP_145907253.1 | iron-containing redox enzyme family protein [*Kitasatospora viridis*] | 91% | 80.53 | 272 |
| WP_266884166.1 | iron-containing redox enzyme family protein [*Streptomyces* sp. NBC_01264] | 99% | 73.94 | 279 |
| MBT2449639.1 | iron-containing redox enzyme family protein [*Streptomyces* sp. ISL-43] | 99% | 71.83 | 279 |
| WP_250738307.1 | iron-containing redox enzyme family protein [unclassified *Streptomyces*] | 99% | 71.83 | 279 |
| WP_251072273.1 | iron-containing redox enzyme family protein [*Streptomyces* sp. ISL-43] | 98% | 72.24 | 278 |
| MBV9139639.1 | iron-containing redox enzyme family protein [*Pseudonocardiales bacterium*] | 94% | 71.48 | 276 |
| WP_014627307.1 | iron-containing redox enzyme family protein [*Streptomycetaceae*] | 93% | 72.18 | 290 |
| CCB73120.1 | conserved protein of unknown function [*Streptantibioticus cattleyicolor* NRRL 8057 = DSM 46488] | 93% | 72.18 | 297 |
| WP_167161217.1 | iron-containing redox enzyme family protein [*Streptomyces* sp. MBT27] | 96% | 72.2 | 278 |
| WP_244927037.1 | iron-containing redox enzyme family protein [*Streptomyces eurocidicus*] | 95% | 71.79 | 277 |
| WP_274550930.1 | iron-containing redox enzyme family protein [*Streptomyces* sp. ZAF1911] | 96% | 72.36 | 279 |
| WP_116214782.1 | iron-containing redox enzyme family protein [*Streptomyces olivoreticuli*] | 92% | 73.58 | 276 |
| MBF6050450.1 | iron-containing redox enzyme family protein [*Streptomyces eurocidicus*] | 95% | 71.79 | 295 |
| WP_301891204.1 | iron-containing redox enzyme family protein [*Streptomyces olivoreticuli*] | 92% | 73.21 | 276 |
| RLU87494.1 | hypothetical protein CTZ27_22980 [*Streptomyces griseocarneus*] | 93% | 71.64 | 278 |
| WP_284715789.1 | iron-containing redox enzyme family protein [*Streptomyces* sp. G1] | 92% | 74.62 | 264 |
| WP_228772874.1 | iron-containing redox enzyme family protein [*Streptomyces eurocidicus*] | 91% | 73.66 | 264 |
| MBT2382898.1 | iron-containing redox enzyme family protein [Streptomyces sp. ISL-11] | 93% | 72.18 | 295 |
| WP_229328797.1 | iron-containing redox enzyme family protein [*Streptomyces* sp. UNOC14_S4] | 92% | 72.83 | 278 |
| WP_189762646.1 | iron-containing redox enzyme family protein [*Streptomyces xanthochromogenes*] | 96% | 71.48 | 278 |
| WP_267754302.1 | iron-containing redox enzyme family protein [*Streptomyces* sp. H34-S4] | 99% | 71.48 | 279 |
| WP_161245663.1 | iron-containing redox enzyme family protein [*Streptomyces* sp. SID1034] | 94% | 72.86 | 278 |
| WP_251018674.1 | iron-containing redox enzyme family protein [*Streptomyces* sp. ISL-11] | 89% | 74.12 | 264 |
| MCP3753520.1 | iron-containing redox enzyme family protein [*Streptomyces* sp. TBY4] | 96% | 71.64 | 279 |
| WP_274815763.1 | iron-containing redox enzyme family protein [*Streptomyces coeruleorubidus*] | 95% | 71.06 | 278 |
| WP_302168684.1 | iron-containing redox enzyme family protein [*Streptomyces* sp. TBY4] | 95% | 72.06 | 278 |
| WP_263207977.1 | iron-containing redox enzyme family protein [*Streptomyces* sp. HUAS 15-9] | 95% | 70.33 | 278 |
| WP_190025862.1 | iron-containing redox enzyme family protein [*Streptomyces xanthochromogenes*] | 94% | 72.12 | 278 |
| WP_267820064.1 | iron-containing redox enzyme family protein [*Streptomyces* sp. H27-H1] | 99% | 70.07 | 279 |
| WP_095581525.1 | iron-containing redox enzyme family protein [*Streptomyces albireticuli*] | 96% | 69.29 | 283 |
| WP_248288569.1 | iron-containing redox enzyme family protein [*Streptomyces albireticuli*] | 94% | 70.48 | 275 |
| WP_232389820.1 | iron-containing redox enzyme family protein [*Streptomyces albireticuli*] | 94% | 70.48 | 287 |
| WP_189509804.1 | iron-containing redox enzyme family protein [*Streptomyces narbonensis*] | 97% | 70.36 | 298 |
| ARZ67274.1 | hypothetical protein SMD11_1613 [*Streptomyces albireticuli*] | 93% | 70.68 | 277 |
| WP_234365957.1 | iron-containing redox enzyme family protein [*Streptomyces albireticuli*] | 89% | 72.37 | 289 |
| WP_267042257.1 | iron-containing redox enzyme family protein [*Streptomyces* sp. NBC_00094] | 98% | 68.77 | 292 |
| WP_055640325.1 | iron-containing redox enzyme family protein [*Streptomyces gardneri*] | 95% | 69.96 | 296 |
| WP_249003486.1 | iron-containing redox enzyme family protein [*Streptomyces* sp. HU2014] | 89% | 71.98 | 291 |
| WP_141293605.1 | iron-containing redox enzyme family protein [*Streptomyces gardneri*] | 94% | 70.11 | 296 |
| WP_189753278.1 | iron-containing redox enzyme family protein [*Streptomyces*] | 96% | 71.12 | 303 |
| WP_030776453.1 | iron-containing redox enzyme family protein [*Streptomyces*] | 97% | 70.5 | 303 |
| WP_249459618.1 | iron-containing redox enzyme family protein [*Streptomyces lavenduligriseus*] | 96% | 70.76 | 303 |
| WDM11072.1 | iron-containing redox enzyme family protein [*Streptomyces lavenduligriseus*] | 96% | 70.76 | 303 |
| WP_181793484.1 | iron-containing redox enzyme family protein [*Streptomyces* sp. WELS2] | 92% | 73.48 | 333 |
| WP_056646699.1 | iron-containing redox enzyme family protein [*Streptomyces* sp. Root431] | 94% | 70.74 | 292 |
| MCX2185255.1 | iron-containing redox enzyme family protein [*Streptomyces* sp. SKN60] | 94% | 70.37 | 305 |
| WP_051753752.1 | iron-containing redox enzyme family protein [*Streptomyces achromogenes*] | 96% | 70.4 | 302 |
| WP_024754983.1 | iron-containing redox enzyme family protein [*Streptomyces exfoliatus*] | 94% | 71.22 | 292 |
| WP_150264407.1 | iron-containing redox enzyme family protein [*Streptomyces venezuelae*] | 94% | 70.74 | 296 |
| MCZ0204274.1 | iron-containing redox enzyme family protein [*Streptomyces* sp. UMAF16] | 96% | 70.4 | 302 |
| WP_125741835.1 | iron-containing redox enzyme family protein [*Streptomyces* sp. WAC01280] | 94% | 70.37 | 292 |
| WP_289967440.1 | iron-containing redox enzyme family protein [*Streptomyces* sp. CSDS2] | 89% | 72.76 | 298 |
| MBV8540531.1 | iron-containing redox enzyme family protein [*Pseudonocardiales bacterium*] | 75% | 77.78 | 219 |
| WP_253835383.1 | iron-containing redox enzyme family protein [*Actinokineospora globicatena*] | 93% | 66.29 | 287 |
| WP_285609844.1 | iron-containing redox enzyme family protein [*Actinokineospora globicatena*] | 93% | 66.29 | 287 |
| WP_121390396.1 | iron-containing redox enzyme family protein [*Actinokineospora cianjurensis*] | 97% | 63.21 | 289 |
| WP_092782860.1 | iron-containing redox enzyme family protein [*Actinokineospora terrae*] | 94% | 64.07 | 288 |
| WP_253884752.1 | iron-containing redox enzyme family protein [*Actinokineospora diospyrosa*] | 94% | 64.07 | 287 |
| WP_211239261.1 | iron-containing redox enzyme family protein [*Actinokineospora inagensis*] | 89% | 63.92 | 269 |
| NUT99080.1 | iron-containing redox enzyme family protein [*Saccharothrix* sp.] | 90% | 59.07 | 281 |

**Table S2** Organic solvent tolerance of SaRohS

| Organic solvents | Relative enzyme activity (%) | |
| --- | --- | --- |
|  | Solvent (20%, v/v) | Solvent (50%, v/v) |
| None (control) | 100 | 100 |
| Methanol | 49 | - |
| Ethanol | 66 | - |
| Acetonitrile | - | - |
| DMSO | 48 | - |
| Dichloromethane | - | - |
| Chloroform | - | - |
| *n*-Butanol | - | - |
| Ethyl acetate | 28 | 10 |

**Table S3** The binding free energy calculated using MM/PBSA

| SaRohS and its mutants | Binding free energy (kcal/mol) |
| --- | --- |
| Wild type | -115.0692 |
| G95A | -269.2814 |
| V112L | -172.8612 |
| L120V | -132.5867 |
| K115T | -133.1530 |
| G95A/K115T | -190.5853 |

**Table S4** Strains, plasmids and primers used in this study

| Strains, plasmids and primers | Description | Source |
| --- | --- | --- |
| *Plasmids* |  |  |
| pETDuet-28a^+^ | pBR322 Kan lacI T7 |  |
| pS1 | pBR322 Kan lacI T7 *aurf* | This study |
| pS2 | pBR322 Kan lacI T7 *cmlI* | This study |
| pS3 | pBR322 Kan lacI T7 *Karohs* | This study |
| pS4 | pBR322 Kan lacI T7 *Sarohs* | This study |
| pS5 | pBR322 Kan lacI T7 *Atrohs* | This study |
| pS5 | pBR322 Kan lacI T7 *Kvrohs* | This study |
| pS6 | pBR322 Kan lacI T7 *Pbrohs* | This study |
| pM1 | pBR322 Kan lacI T7 *Sarohs^G95A^* | This study |
| pM2 | pBR322 Kan lacI T7 *Sarohs^V112L^* | This study |
| pM3 | pBR322 Kan lacI T7 *Sarohs^L120V^* | This study |
| pM4 | pBR322 Kan lacI T7 *Sarohs^M168F^* | This study |
| pM5 | pBR322 Kan lacI T7 *Sarohs^L205Q^* | This study |
| pM6 | pBR322 Kan lacI T7 *Sarohs^A216R^* | This study |
| pM7 | pBR322 Kan lacI T7 *Sarohs^L232M^* | This study |
| pM8 | pBR322 Kan lacI T7 *Sarohs^A262G^* | This study |
| pM9 | pBR322 Kan lacI T7 *Sarohs^T75A^* | This study |
| pM10 | pBR322 Kan lacI T7 *Sarohs^S198D^* | This study |
| pM11 | pBR322 Kan lacI T7 *Sarohs^S198Y^* | This study |
| pM12 | pBR322 Kan lacI T7 *Sarohs^K115R^* | This study |
| pM13 | pBR322 Kan lacI T7 *Sarohs^K115T^* | This study |
| pM14 | pBR322 Kan lacI T7 *Sarohs^L212V^* | This study |
| pM15 | pBR322 Kan lacI T7 *Sarohs^L212I^* | This study |
| pM16 | pBR322 Kan lacI T7 *Sarohs^D266K^* | This study |
| pM17 | pBR322 Kan lacI T7 *Sarohs^D266A^* | This study |
| pM18 | pBR322 Kan lacI T7 *Sarohs^G95A/V112L^* | This study |
| pM19 | pBR322 Kan lacI T7 *Sarohs^G95A/L120V^* | This study |
| pM20 | pBR322 Kan lacI T7 *Sarohs^G95A/K115T^* | This study |
| pM21 | pBR322 Kan lacI T7 *Sarohs^V112L/K115T^* | This study |
| pM22 | pBR322 Kan lacI T7 *Sarohs^K115T/L120V^* | This study |
| pM23 | pBR322 Kan lacI T7 *Sarohs^V112L/L120V^* | This study |
| *Strains* |  |  |
| *E. coli* DH5 | F− *rec*A *endA1 Φ80dlacZΔM15 hsdR17*(*r_K_^−^ m_K_^+^*)*λ^−^* | Invitrogen |
| *E. coli* BL21 (DE3) | F− *ompT hsdS_B_ (r_B_^−^ m_B_^−^) gal dcm* λ (DE3) | Invitrogen |
| F092 | *E.coli* BL21(DE3) carrying pS1 | This study |
| F093 | *E.coli* BL21(DE3) carrying pS2 | This study |
| F089 | *E.coli* BL21(DE3) carrying PS3 | This study |
| F124 | *E.coli* BL21(DE3) carrying pS4 | This study |
| F125 | *E.coli* BL21(DE3) carrying pS5 | This study |
| F223 | *E.coli* BL21(DE3) carrying pS6 | This study |
| F224 | *E.coli* BL21(DE3) carrying pS7 | This study |
| F135 | *E.coli* BL21(DE3) carrying pM1 | This study |
| F136 | *E.coli* BL21(DE3) carrying pM2 | This study |
| F137 | *E.coli* BL21(DE3) carrying pM3 | This study |
| F138 | *E.coli* BL21(DE3) carrying pM4 | This study |
| F139 | *E.coli* BL21(DE3) carrying pM5 | This study |
| F140 | *E.coli* BL21(DE3) carrying pM6 | This study |
| F147 | *E.coli* BL21(DE3) carrying pM7 | This study |
| F148 | *E.coli* BL21(DE3) carrying pM8 | This study |
| F149 | *E.coli* BL21(DE3) carrying pM9 | This study |
| F150 | *E.coli* BL21(DE3) carrying pM10 | This study |
| F151 | *E.coli* BL21(DE3) carrying pM11 | This study |
| F152 | *E.coli* BL21(DE3) carrying pM12 | This study |
| F153 | *E.coli* BL21(DE3) carrying pM13 | This study |
| F154 | *E.coli* BL21(DE3) carrying pM14 | This study |
| F155 | *E.coli* BL21(DE3) carrying pM15 | This study |
| F156 | *E.coli* BL21(DE3) carrying pM16 | This study |
| F157 | *E.coli* BL21(DE3) carrying pM17 | This study |
| F205 | *E.coli* BL21(DE3) carrying pM18 | This study |
| F206 | *E.coli* BL21(DE3) carrying pM19 | This study |
| F207 | *E.coli* BL21(DE3) carrying pM20 | This study |
| F208 | *E.coli* BL21(DE3) carrying pM21 | This study |
| F209 | *E.coli* BL21(DE3) carrying pM22 | This study |
| F210 | *E.coli* BL21(DE3) carrying pM23 | This study |
| *Primers* | *Sequence* |  |
| G95A-F | AAACACGCGCTTAAACGGGACCTACCG |  |
| G95A-R | CGTTTAAGCGCGTGTTTTGACGCTTTG |  |
| V112L-F | GGCCGCCACTACGCGAACGGCTAATT |  |
| V112L-R | TCGCGTAGTGGCGGCCAAAAAAATTC |  |
| L120V-F | TTTTCGGTTTACCGCCACTACGCGCAC |  |
| L120V-R | GGCGGTAAACCGAAAATTAGCCGTGC |  |
| M168F-F | CTACTTTGGAAAGTCAAGGACTTACGCAAA |  |
| M168F-R | CTTGACTTTCCAAAGTAGCGACCACATAG |  |
| L205Q-F | TCGCGCAAATCGACGGTCCGCGTTCGC |  |
| L205Q-R | ACCGTCGATTTGCGCGAGCCTTATGAG |  |
| A216R-F | CCAAACCTTTTACCGTCCATAATATGG |  |
| A216R-R | GACGGTAAAAGGTTTGGGTCCGCCGATG |  |
| L232M-F | AAGGTGTACTTTCTCTACGTCTGGCG |  |
| L232M-R | AGAGAAAGTACACCTTGCGTAGTTAT |  |
| A262G-F | GACTCGTGGGACTAACCCCCACGCCAC |  |
| A262G-R | GGTTAGTCCCACGAGTCGTCAAAGCGC |  |
| T75A-F | GAAAGCGCCCGCTTTTTAGCGGAAGATGCGAC |  |
| T75A-R | AAAAAGCGGGCGCTTTCGCTGCCCGCCGCTTC |  |
| K115R-F | GCCGATTAGAAGCCAAATGGCGCTGATGCGCG |  |
| K115R-R | TTTGGCTTCTAATCGGCACGCGCATCACCGCC |  |
| K115T-F | GCCGATTACAAGCCAAATGGCGCTGATGCGCG |  |
| K115T-R | TTTGGCTTGTAATCGGCACGCGCATCACCGCC |  |
| S198D-F | GTATTCCGGACGCGTTTAGCTGCCTGGCGCAAG |  |
| S198D-R | TAAACGCGTCCGGAATACTCGCTTCCAGATACG |  |
| S198Y-F | GTATTCCGTACGCGTTTAGCTGCCTGGCGCAAG |  |
| S198Y-R | TAAACGCGTACGGAATACTCGCTTCCAGATACG |  |
| L212V-F | GCCTGGGTGTGGAAAATGGCGCGTATTATACC |  |
| L212V-R | ATTTTCCACACCCAGGCGGCTACACGCTTGCG |  |
| L212I-F | CGCCTGGGTATTGAAAATGGCGCGTATTATACCG |  |
| L212I-R | TTTTCAATACCCAGGCGGCTACACGCTTGCGC |  |
| D266K-F | CGGTGAAAGCAGCGGTTGCGAGAGCGCGTCGT |  |
| D266K-R | AACCGCTGCTTTCACCGCACCCGCAATCAGGG |  |
| D266A-F | CTGCAGCGGTTGCGAGAGCGCGTCGTCAAGTT |  |
| D266A-R | TCTCGCAACCGCTGCAGCCACCGCACCCGCAATCA |  |
